# Supplementary material for: HemoDownloader: Open source software utility to extract data from HemoCue HbA1c 501 devices in epidemiological studies of diabetes mellitus
Source: PLoS One. 2020 Nov 17;15(11):e0242087. doi: 10.1371/journal.pone.0242087 (PMC7671527; doi:10.1371/journal.pone.0242087)
Supplement: S1 File — (ZIP) [file pone.0242087.s001.zip › src/dependencies/XlsxWriter-1.1.8/docs/readme.html]

Getting Started with XlsxWriter — XlsxWriter Readme


### Navigation

- XlsxWriter Readme »

# Getting Started with XlsxWriter

Here are some easy instructions to get you up and running with the XlsxWriter
module.

## Installing XlsxWriter

The first step is to install the XlsxWriter module. There are several ways to
do this.

### Using PIP

The pip installer is
the preferred method for installing Python modules from
PyPI, the Python Package Index:

```
$ sudo pip install XlsxWriter
```

Note

Windows users can omit sudo at the start of the command.

### Using Easy\_Install

If pip doesn’t work you can try
easy\_install:

```
$ sudo easy_install install XlsxWriter
```

### Installing from a tarball

If you download a tarball of the latest version of XlsxWriter you can install
it as follows (change the version number to suit):

```
$ tar -zxvf XlsxWriter-1.2.3.tar.gz

$ cd XlsxWriter-1.2.3
$ sudo python setup.py install
```

A tarball of the latest code can be downloaded from GitHub as follows:

```
$ curl -O -L http://github.com/jmcnamara/XlsxWriter/archive/master.tar.gz

$ tar zxvf master.tar.gz
$ cd XlsxWriter-master/
$ sudo python setup.py install
```

### Cloning from GitHub

The XlsxWriter source code and bug tracker is in the
XlsxWriter repository on GitHub.
You can clone the repository and install from it as follows:

```
$ git clone https://github.com/jmcnamara/XlsxWriter.git

$ cd XlsxWriter
$ sudo python setup.py install
```

## Running a sample program

If the installation went correctly you can create a small sample program like
the following to verify that the module works correctly:

```
import xlsxwriter

workbook = xlsxwriter.Workbook('hello.xlsx')
worksheet = workbook.add_worksheet()

worksheet.write('A1', 'Hello world')

workbook.close()
```

Save this to a file called hello.py and run it as follows:

```
$ python hello.py
```

This will output a file called hello.xlsx which should look something like
the following:

If you downloaded a tarball or cloned the repo, as shown above, you should also
have a directory called
examples
with some sample applications that demonstrate different features of
XlsxWriter.

# Documentation

The full version of XlsxWriter documentation is hosted on
Read The Docs. It is
also available as a
PDF.

© Copyright 2013-2019, John McNamara.
