# Supplementary figures and images for: HemoDownloader: Open source software utility to extract data from HemoCue HbA1c 501 devices in epidemiological studies of diabetes mellitus
Source: PLoS One. 2020 Nov 17;15(11):e0242087. doi: 10.1371/journal.pone.0242087 (PMC7671527; doi:10.1371/journal.pone.0242087)

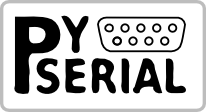

Supplement: S1 File — (ZIP) [file pone.0242087.s001.zip › src/dependencies/pyserial-3.4/documentation/pyserial.png]

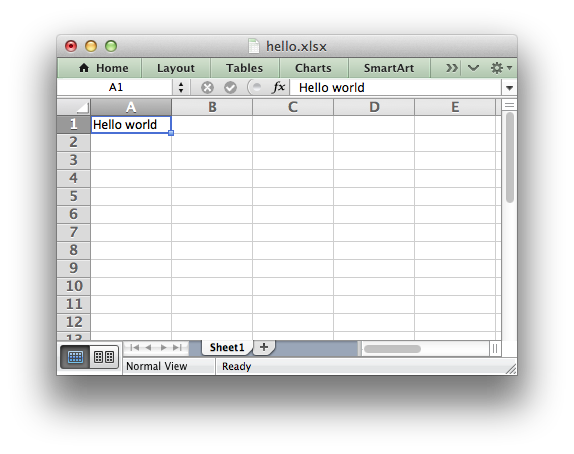

Supplement: S1 File — (ZIP) [file pone.0242087.s001.zip › src/dependencies/XlsxWriter-1.1.8/docs/_static/hello01.png]

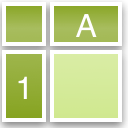

Supplement: S1 File — (ZIP) [file pone.0242087.s001.zip › src/dependencies/XlsxWriter-1.1.8/docs/_static/logo.png]
